# Monitoring biological water quality by volunteers complements professional assessments

**DOI:** 10.1371/journal.pone.0263899

**Published:** 2022-02-25

**Authors:** Edwin T. H. M. Peeters, Anton A. M. Gerritsen, Laura M. S. Seelen, Matthijs Begheyn, Froukje Rienks, Sven Teurlincx

**Affiliations:** 1 Chairgroup Aquatic Ecology and Water Quality Management, Wageningen University, Wageningen, The Netherlands; 2 De Waterspin Advies, Arnhem, The Netherlands; 3 Department of Planning and Monitoring, Regional Water Authority Brabantse Delta, Breda, The Netherlands; 4 Global Learning and Observations to Benefit the Environment (GLOBE) Netherlands, Utrecht, The Netherlands; 5 Section Public Relations & Science Communication, Netherlands Institute of Ecology (NIOO-KNAW), Wageningen, The Netherlands; 6 Department of Aquatic Ecology, Netherlands Institute of Ecology (NIOO-KNAW), Wageningen, The Netherlands; University of Maine, UNITED STATES

## Abstract

Progressively more community initiatives have been undertaken over last decades to monitor water quality. Biological data collected by volunteers has been used for biodiversity and water quality studies. Despite the many citizen science projects collecting and using macroinvertebrates, the number of scientific peer-reviewed publications that use this data, remains limited. In 2018, a citizen science project on biological water quality assessment was launched in the Netherlands. In this project, volunteers collect macroinvertebrates from a nearby waterbody, identify and count the number of specimens, and register the catch through a web portal to instantaneously receive a water quality score based on their data. Water quality monitoring in the Netherlands is traditionally the field of professionals working at water authorities. Here, we compare the data from the citizen science project with the data gathered by professionals. We evaluate information regarding type and distribution of sampled waterbodies and sampling period, and compare general patterns in both datasets with respect to collected animals and calculated water quality scores. The results show that volunteers and professionals seldomly sample the same waterbody, that there is some overlap in sampling period, and that volunteers more frequently sampled urban waters and smaller waterbodies. The citizen science project is thus yielding data about understudied waters and this spatial and temporal complementarity is useful. The character and thoroughness of the assessments by volunteers and professionals are likely to differentiate. Volunteers collected significantly lower numbers of animals per sample and fewer animals from soft sediments like worms and more mobile individuals from the open water column such as boatsmen and beetles. Due to the lack of simultaneous observations at various locations by volunteers and professionals, a direct comparison of water quality scores is impossible. However, the obtained patterns from both datasets show that the water quality scores between volunteers and professionals are dissimilar for the different water types. To bridge these differences, new tools and processes need to be further developed to increase the value of monitoring biological water quality by volunteers for professionals.

## Introduction

Although there are various levels of involvement of volunteers in science projects [1, 2], most citizen science projects focus on generating data and information about the quality of their surrounding environment. As such, volunteers can contribute to the more traditional way of environmental monitoring by professionals resulting in a combined database of greater temporal and spatial scale [3, 4]. According to the UN, citizen science will be crucial for monitoring the progress of a number of the Sustainable Development Goals (SDG) [5, 6]. Furthermore, the collected data through volunteers will also become essential in the UN Sustainable Development Goals reporting [7]. Among others, SDG 6 (clean water and sanitation) is expected to greatly benefit from volunteer participation [8, 9]. Additionally, the European Water Framework Directive encourages the involvement of interested parties in the implementation of the Directive [10, 11] and among the EU citizens there are many that want to provide input, expertise, time and even money to help protect fresh waters [12].

Observations on the natural environment have been recorded by amateurs already for a very long time [13, 14]. The attention for this kind of observations has increased enormously in the last decades as a result of technical developments that facilitated communication among involved partners and lowered the costs of required equipment for data collection [15]. Within the field of ecology, observations by non-trained volunteers on e.g. distribution and phenology of animals and plants have been performed for centuries [13]. Most biological observations by volunteers contribute to research related to biodiversity and species richness [4]. While volunteers can contribute valuable data, trained observers are required for identifying specimens to the species or genus level [16], a level that is needed for most biodiversity investigations. Interestingly, several examples show that biodiversity inventories made by volunteers can impact policy and decision making [17].

In the field of water quality, multiple citizen science projects have been set up and carried out. Several projects were developed to monitor abiotic conditions in waterbodies such as nutrients in urban waterways [18] or in agricultural streams [19], oxygen in streams [20], hydrology [21], colour of water [22], bacteriological water quality [23] and microplastics [24] or a combination of different abiotic variables [25–27]. The first use of aquatic macroinvertebrates in a volunteer monitoring program probably dates back to the mid 1970s [28]. Anglo-Saxon countries in particular, regularly include macroinvertebrates in volunteer monitoring programs for example, approximately 50% of the 1700 volunteer water monitoring programs in the USA (http://volunteermonitoring.org/) (personal comment K. Stepenuck), in the British Anglers’ Riverfly Monitoring Initiative [29], and in the New-Zealand Wai Care program [30]. Although scientific publications exist that make use of data collected by volunteers [e.g. [29, 31–33], their number is still limited.

More than a century ago Kolkwitz & Marsson [34, 35] developed the concept of bioindicators for assessing the quality of water. Macroinvertebrates have been regarded as good bioindicators among others because they occur worldwide in different water systems, are rich in species with much variation in their response to environmental conditions, integrate effects of multiple stressors and integrate effects of stressors over time [36–38]. Several biotic indices have been developed in which the presence of bioindicators was used to assess water quality with and without combination of a measure of species richness such as the Trent Biotic Index (TBI) [39], the Biological Monitoring Working Party (BMWP) [40], the Hilsenhoff biotic index [41], the Belgian Biotic Index (BBI) [42], or the South African Scoring System (SASS) [43]. However, changes in environmental conditions are reflected in a changed composition of the whole macroinvertebrate community and not only in the presence or absence of the bioindicators [44]. The use of the relative abundance of macroinvertebrates is, therefore, considered more sensitive to subtle changes in water quality. As a result, other assessment tools have been developed that included the relative amount or abundance of indicators e.g. [44–47]. The use of macroinvertebrates as a tool for the assessment of water quality has been much more applied in running waters than in standing waters (lakes & ponds) where phytoplankton has been the most important biological group to evaluate the biological quality. Macroinvertebrate assessment tools that can be applied to multiple freshwater types are very scarce.

In many countries, monitoring biological or ecological water quality is legally tasked to water authorities. In the Netherlands, regional water authorities have a long tradition in collecting data on plant and animal life and abiotic conditions in waterbodies. The Dutch water authorities are responsible for the surface water quality in their respective management areas and are responsible to determine, maintain and improve the water quality in regards to the European Water Framework Directive (WFD) [11]. A nationally determined and accepted methodology is used to classify a water system from excellent to very bad upon macroinvertebrate species and abundances, tailored to water type (e.g. river, small lake or ditch). For this WFD mandated monitoring, locations are chosen to represent larger watersheds and timing of sampling is in accordance with the guidelines. Accredited labs that supply the water authorities with these data implement the national quality assurance protocol to assure high quality of data. These data are then used for assessment and reporting of water quality to the EU in accordance with the EU Water Framework Directive guidelines [48]. The collected information is stored in large databases and made publicly available.

In 2018, a citizen science project on biological water quality assessment by means of macroinvertebrates was launched in the Netherlands. The purpose of this project was to engage citizens in water quality and to obtain a nationwide overview of the water quality in the various water types based on data provided by volunteers. Identifying water animals at the species level is not easy and therefore, a biological assessment tool for the general public cannot rely on the level of identification as applied by experts. To involve volunteers in biological water quality assessment, the level of identification should be limited [31, 49] as is, for example, the case with the British Freshwater name trail [50]. This name trail lists more than 40 taxa on a chart together with clues to identify them and is highly suitable for untrained persons. In the Dutch project, volunteers or school classes may collect macroinvertebrates in a nearby surface water, identify and count the number of specimens, and register the catch at the website ‘Waterdiertjes.nl’. It is suggested to collect minimally 50 individuals. To receive as much contributions from volunteers as possible no strict guidelines or regulations regarding sampling have been provided but a very general description is available. Once a volunteer has uploaded the list of species with associated counts, the web application calculates a water quality score based on the information provided and sends this back to the volunteer as a reward. Furthermore, new assessments are together with all available assessments immediately displayed on a map covering the whole country. A small team of experts regularly evaluates the provided data by checking geographical positions, provided written texts, and listed counts of collected animals. In case a sampling is considered strange, it receives a flag and is no longer displayed on the map. Since the project has been running for 3 years now, a considerable amount of data is available for a comparison with data from professionals. Such comparative analyses have been performed previously for, among others, mosquitos in Germany [51], marine debris [52], attributes of vegetation [53], corals [54] and chemical water quality [18, 55, 56], but are relatively scarce for biological water quality monitoring [31]. Here, we compare data collected by volunteers in an unstructured way with those collected according to quality protocols by professionals employed at regional water authorities by focussing on (1) the spatial and temporal distribution of the samplings, (2) the investigated water types and patterns in both datasets with respect to (3) calculated water quality scores and (4) the sampled macroinvertebrate communities. Such a comparison might on the one hand show complementarity and the benefit for professionals of using volunteer data and on the other hand might provide clues to improve the quality of the volunteer data which may thereafter become beneficial for the monitoring by the volunteers. We also present the tool that volunteers use for assessing the biological water quality.

## Material and methods

### Data selection for present study

The data collected by volunteers by scooping and sorting in the period 2018–2020 was retrieved from the website Waterdiertjes.nl and included at least the geographical position of the sampling, date, user-ID, counts of the macroinvertebrate taxa, and water type. The list of included macroinvertebrate taxa is presented in Table 1.

**Table 1:** Overview of taxa used in Waterdiertjes.nl with their level of identification, their water quality indicator value (1 = very bad, 2 = poor, 3 = moderate, 4 = good, 5 = excellent), preferred habitat indicated by the greyish coloured cell, and their degree of mobility (1 = slow; 2 = moderate, 3 = fast).

Latin name	Identification level	Water Quality Indication*	Habitat^$^				Mobility^#^ class
			Solid substrate	Soft sediment	Open water column	Water surface	
Ancylidae	Family	3					1
Anisoptera	subOrder	5					2
Argyroneta	Genus	4					2
Asellidae	Family	2					2
Astacidae	Family	3					3
Baetidae	Family	4					3
Bivalvia	Class	3					1
Calopteryx	Genus	5					2
Ceratopogonidae	Family	3					2
Chaoboridae	Family	3					2
Chironomidae	Family	3					2
Coleoptera	Order	4					3
Collembola	Class	3					1
Corixidae	Family	4					3
Culicidae	Family	1					1
Dixidae	Family	3					1
Ephemerellidae	Family	5					3
Eristalis	Genus	1					1
Gammaridae	Family	5					3
Gerridae	Family	4					2
Gyrinidae	Family	5					2
Heptageniidae	Family	5					3
Hirudinea	subClass	2					1
Hydracarina	Class	3					2
Hydrometridae	Family	5					2
Lymnaeidae	Family	1					1
Nematomorpha	Phylum	4					1
Nepa	Genus	3					1
Notonectidae	Family	4					3
Oligochaeta	subClass	1					1
Planorbidae	Family	1					1
Plathelminthes	Phylum	3					1
Plecoptera	Order	5					2
Ranatra	Genus	3					1
Sialidae	Family	4					1
Simuliidae	Family	5					1
Tipulidae	Family	3					1
Trichoptera	Order	4					1
Veliidae	Family	5					2
Zygoptera	subOrder	4					2

^$^ Based on Verdonschot PFM (1990) Ecological characterization of surface waters in the province of Overijssel (The Netherlands). PhD-thesis Wageningen University, The Netherlands.

^#^ Based on Usseglio-Polatera P, Bournaud M, Richoux P, Tachet H (2000) Biological and ecological traits of benthic freshwater macroinvertebrates: relationships and definition of groups with similar traits. Freshw Biol 43:175–205.

Data from professionals is stored on the freely available website ‘waterkwaliteitsportaal.nl’ and included the geographical position of the sampling, date, abundance of macroinvertebrates, and a description of water type. Only macroinvertebrate recordings from the period 2013–2019 were retrieved that were collected following the multihabitat approach [57] with a standardized sampling length of 5 m. Furthermore, the geographical information from 2011–2019 was also retrieved. Since the classification of water types used by the professionals was more detailed, this typology was adjusted to the one from the citizen science project (Table 2). Also, the taxonomic level of identification by the professionals was adjusted to the one used in the citizen science project (see Table 1).

**Table 2:** Overview of water types used in the citizen science project Waterdiertjes.nl.

Urban		Rural	
Linear	Oval shape	Linear	Oval shape
City pond	City canal	Stream	Pond (Pond)
Garden pond		River	Lake
		Ditch	
		Canal	

### Data analyses

#### General characteristics of professional and citizen science data

The mean yearly number of observations was determined for both datasets to compare the efforts of both volunteers and professionals. The degree of participation in the citizen science project was determined by counting the number of unique user-IDs. Since season may be important for water quality assessment, the number of samples per month were calculated and compared between professionals and volunteers.

Available coordinates of the locations from both the professionals and the volunteers were used to calculate the distances between a citizen science location and all professional locations and the shortest distance was determined. For this analysis, all citizen science locations were used and all locations visited by the professionals between 2011 and 2019 because various authorities have a 3 years cycling monitoring program. A frequency distribution of these shortest distances was made.

To investigate whether volunteers sample different water types than professionals, the number of samples per water type were counted and compared between professionals and volunteers. Fisher’s Exact Test [58] was used to test whether differences in observed frequencies were significant.

#### Characteristics of collected macroinvertebrates

Mean number of individuals per sample caught was calculated for both datasets. A Generalized Linear Model (GLM) [59] with total number of individuals per sample as dependent variable and collector, professional or volunteers, as independent variable and with a Poisson distribution and log link was used to evaluate the significance of this difference.

For each taxon, the main habitat of occurrence (soft sediment, solid substrate, open water column, water surface) was determined using [60] and taxa were classified after their mobility in classes (slow, moderate, fast) using [61] (Table 1). Total number of individuals per habitat and per mobility class were determined as well as the relative distribution. Fisher’s Exact Tests [58] were used to evaluate whether the distribution over the habitat and mobility classes were significantly different between the citizen science and professional data.

#### Biological water quality assessment by volunteers

Based on the macroinvertebrate taxa present on the British Macrofauna name trail [50], a biological assessment system has been developed that should be applicable in all kinds of freshwaters in the Netherlands. Therefore, each macroinvertebrate taxon was assigned a water quality score (Table 1). These scores and the counts are used to calculate the overall (weighted average) Citizen Science Water Quality (WQ_cs_) sample score:

$$WQcs=\frac{\sum_{i=1}^{5}i\cdot n_{i}}{\sum n_{i}}$$

With *n*_*i*_ = number of individuals belonging to water quality class i

*i* = water quality indication with 1 = very bad; 2 = poor; 3 = moderate, 4 = good; 5 = excellent

The WQ_cs_ score ranges between 1 and 5 and this range is divided in five quality classes, being very bad (score 1–1.8), poor (1.8–2.6), moderate (2.6–3.4), good (3.4–4.2) and excellent (4.2–5.0).

A web application was developed in which both the taxa from the British Freshwater name trail [50] and the score for water quality were included (https://waterdiertjes.nl). Each macroinvertebrate taxon is represented by a drawing in the WebApp and counts of the collected animals can be registered per taxon. When all collected animals have been entered, the total list of recordings can be uploaded together with additional information like geographical location and water type. Immediately thereafter, the WQ_sc_ will appear on the screen. Data is stored in a database which is freely accessible and available.

The WQ_sc_ was also calculated for the data obtained by the professionals for the period 2013–2019. In order to find out whether the frequency distributions over the quality classes were significantly different, Fisher’s Exact Tests [58] were applied per water type.

All analyses were performed in IBM SPSS Statistics 25.

## Results

### General characteristics of professional and citizen science data

In the period 2018–2020, volunteers collected macroinvertebrates in 1646 occasions (on average 550 per year). In total 1339 unique locations were sampled. Based on unique user-IDs, a maximum number of 300, 322 and 586 volunteers participated in 2018, 2019 and 2020 respectively. A total of 1150 unique user-IDs (individuals and/or small groups) supplied data over the three years. Professionals from regional water authorities collected 6103 samples in freshwater ecosystems in the period 2013–2019 with more than 1000 samples per year in the last four years.

**Fig 1:**
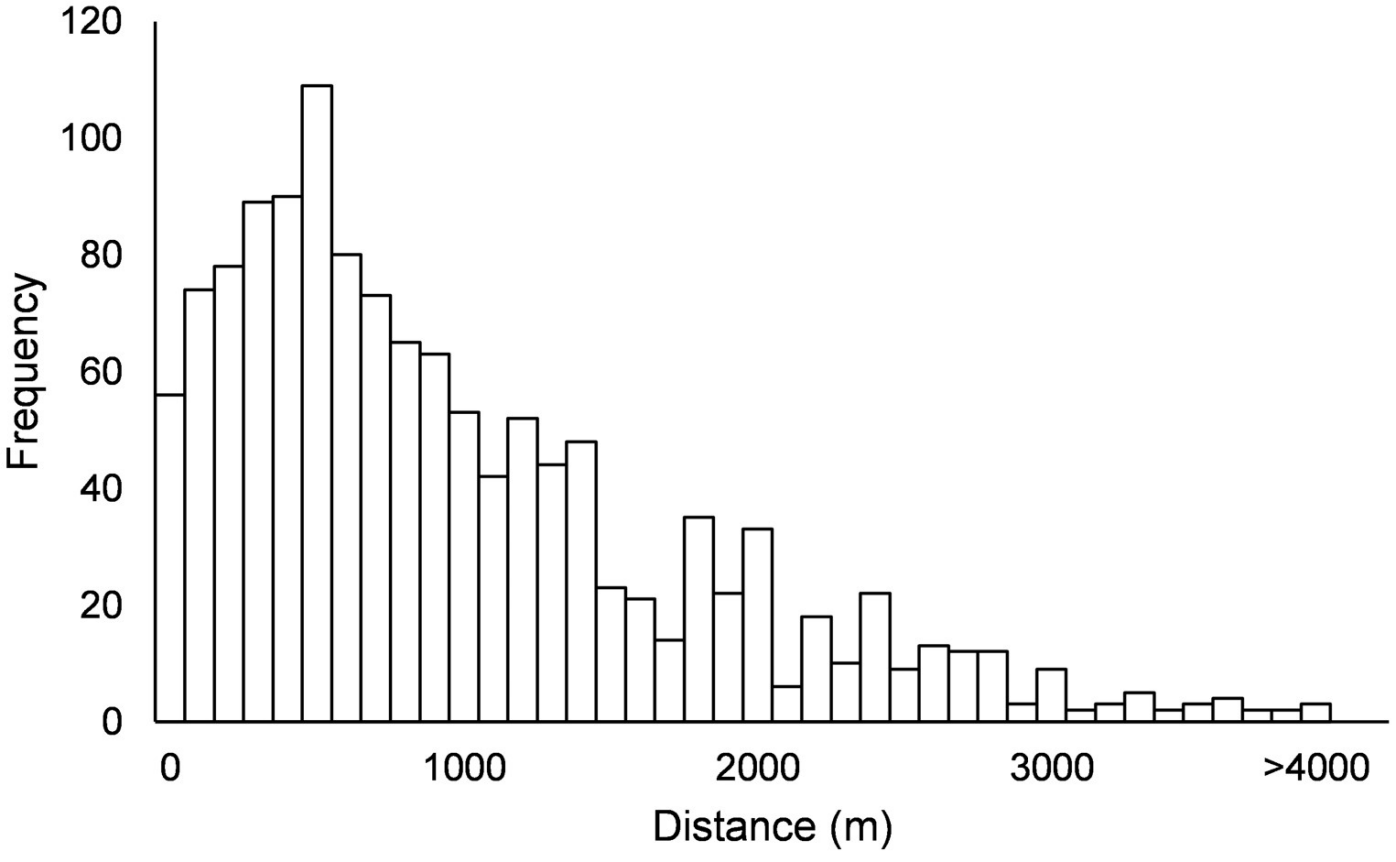
Frequency distribution of closest distance between a sampled location by volunteers and sampling points from professionals in the period 2011–2019 (n = 1304).

The majority of the locations sampled by volunteers was within 1 km distance from locations sampled by professionals (Fig 1) and the maximum distance was 5.5 km. Only a tiny fraction (n = 32) of the volunteers’ locations was in real close vicinity (< 50 m) of locations sampled by professionals.

**Fig 2:**
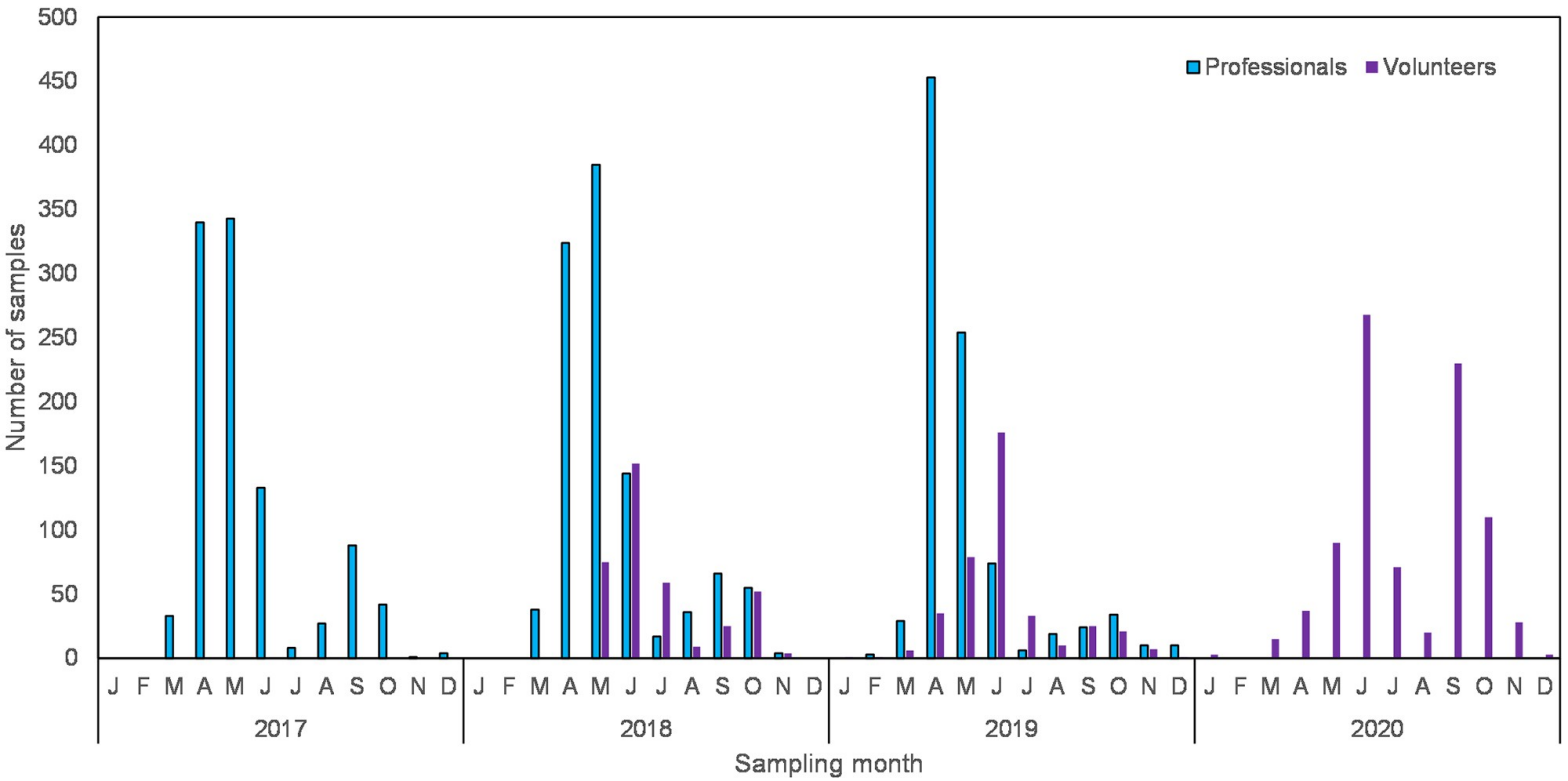
Number of samples per month taken by professionals and volunteers. Since the pattern for the professionals was quite stable only the last three years (2017–2019) were plotted.

The total number of samples per month varied largely throughout the year and this pattern was quite stable for the professionals with the majority of samples taken in April and May (Fig 2). Volunteers collected most samples in May and June but also in September and October in 2020 (Fig 2).

**Fig 3:**
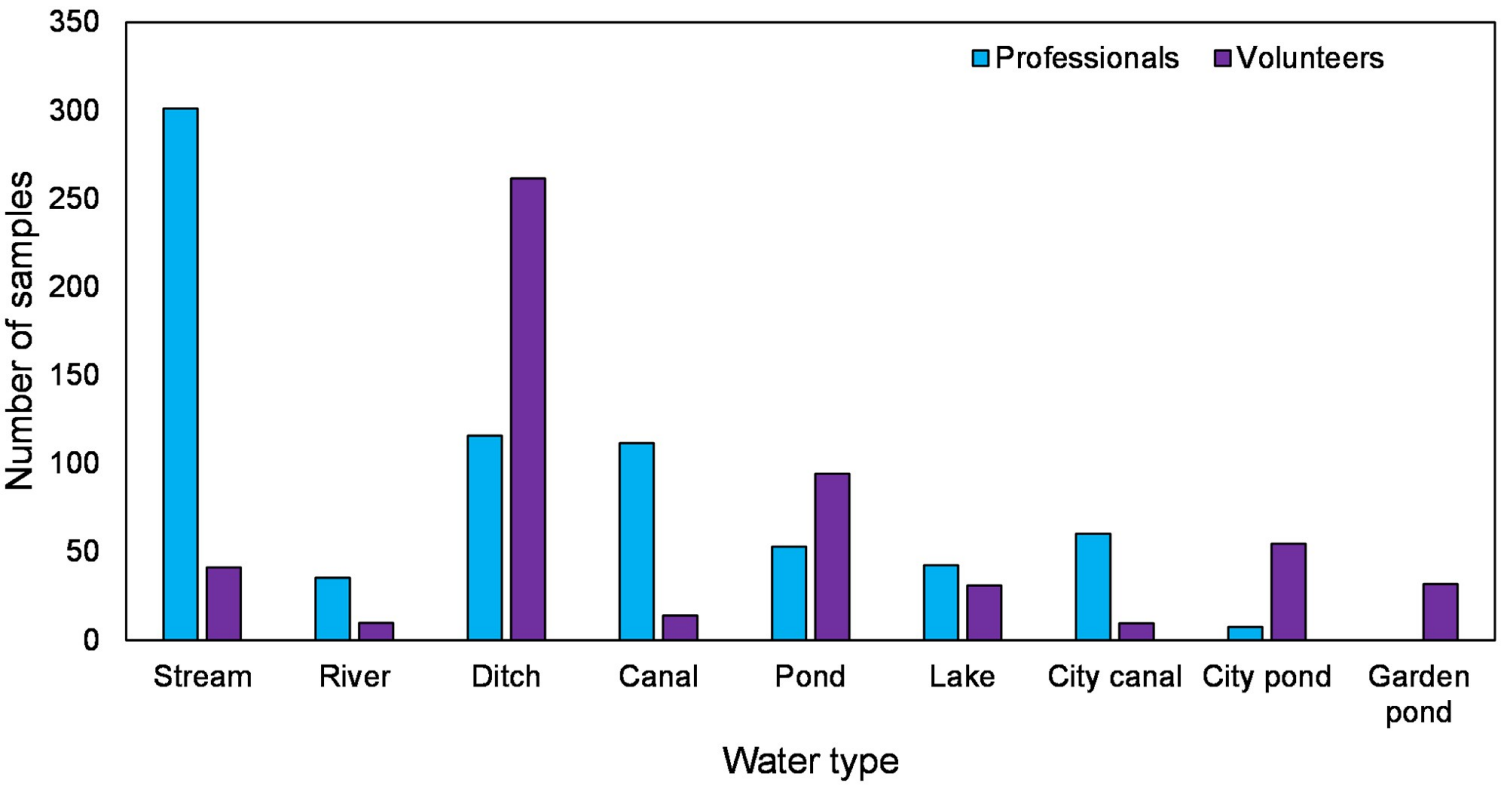
Yearly average number of samples taken by professionals (2013–2019) and volunteers (2018–2020) per water type. Information on water type was missing for roughly 12% of the professional locations.

Volunteers took their samples primarily in ditches, ponds & lakes and urban waters while flowing waters and canals were underrepresented (Fig 3). In contrast, professionals sampled mostly in streams, ditches and canals. Observations by professionals in the urban environment were very limited. City and garden ponds were hardly sampled by professionals while volunteers sampled these relatively small systems much more frequently. The distribution of the samples over the different water types is significantly different between professionals and volunteers. A Fisher’s Exact Test showed that for all water types the number of observations differed significantly (Chi-Square = 443.955, df = 8, p ≤ 0.001) between professionals and volunteers except for lakes (SI Appendix in S1 File).

### Characteristics of collected macroinvertebrates

Volunteers collected less individuals from the included taxa per sample than professionals (on average 61 and 1145 respectively) and this difference was significant (GLM: Wald Chi-Square = 851816.23, d.f. = 1, p<0.001).

**Fig 4:**
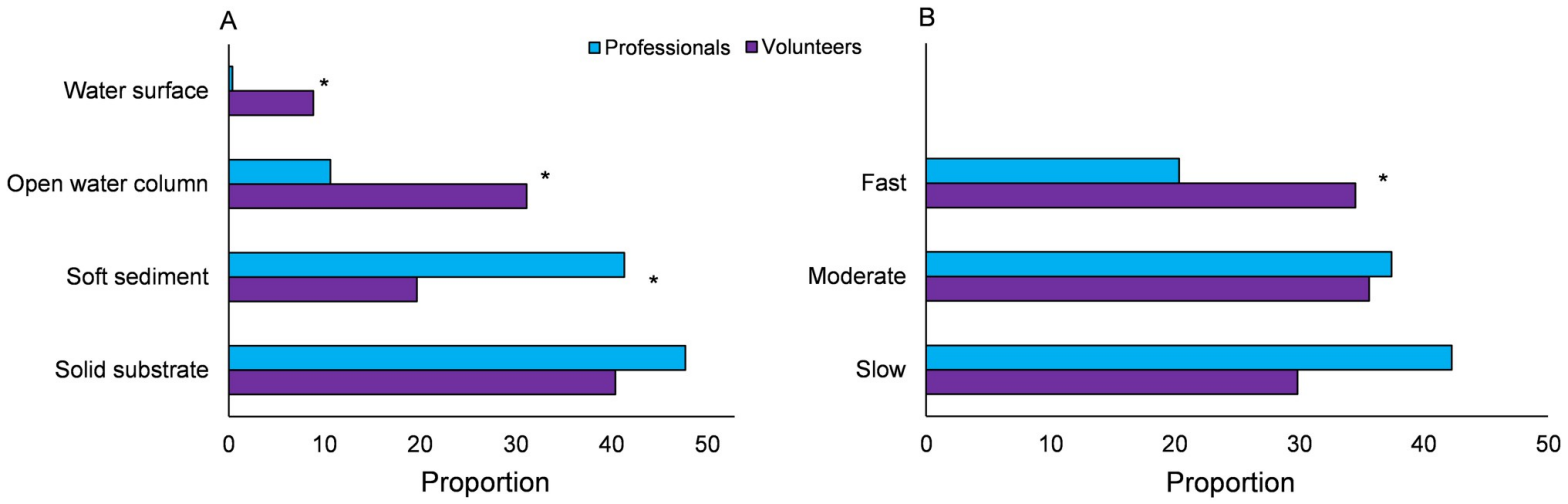
Relative distribution of caught animals by volunteers and professionals over A) different habitats and B) mobility classes. An * indicates that the difference is significant.

Professionals caught more animals that inhabit soft sediments than volunteers who reported much more animals from the open water column or surface of the waterbodies in their samples (Fig 4A). A Fisher’s Exact Test based on the percentage distribution over the habitats showed that all differences between professionals and volunteers were significant except for solid substrate (Chi-square 27.495; df = 3; p<0.001). Volunteers also had relatively more mobile specimens in their samples than professionals (Fig 4B). A Fisher’s Exact Test based on the distribution over the mobility classes showed that especially the differences between the proportion of fast-moving animals was significant (Chi-squared 6.063; df = 2; p = 0.049).

### Biological water quality assessment by volunteers

**Fig 5:**
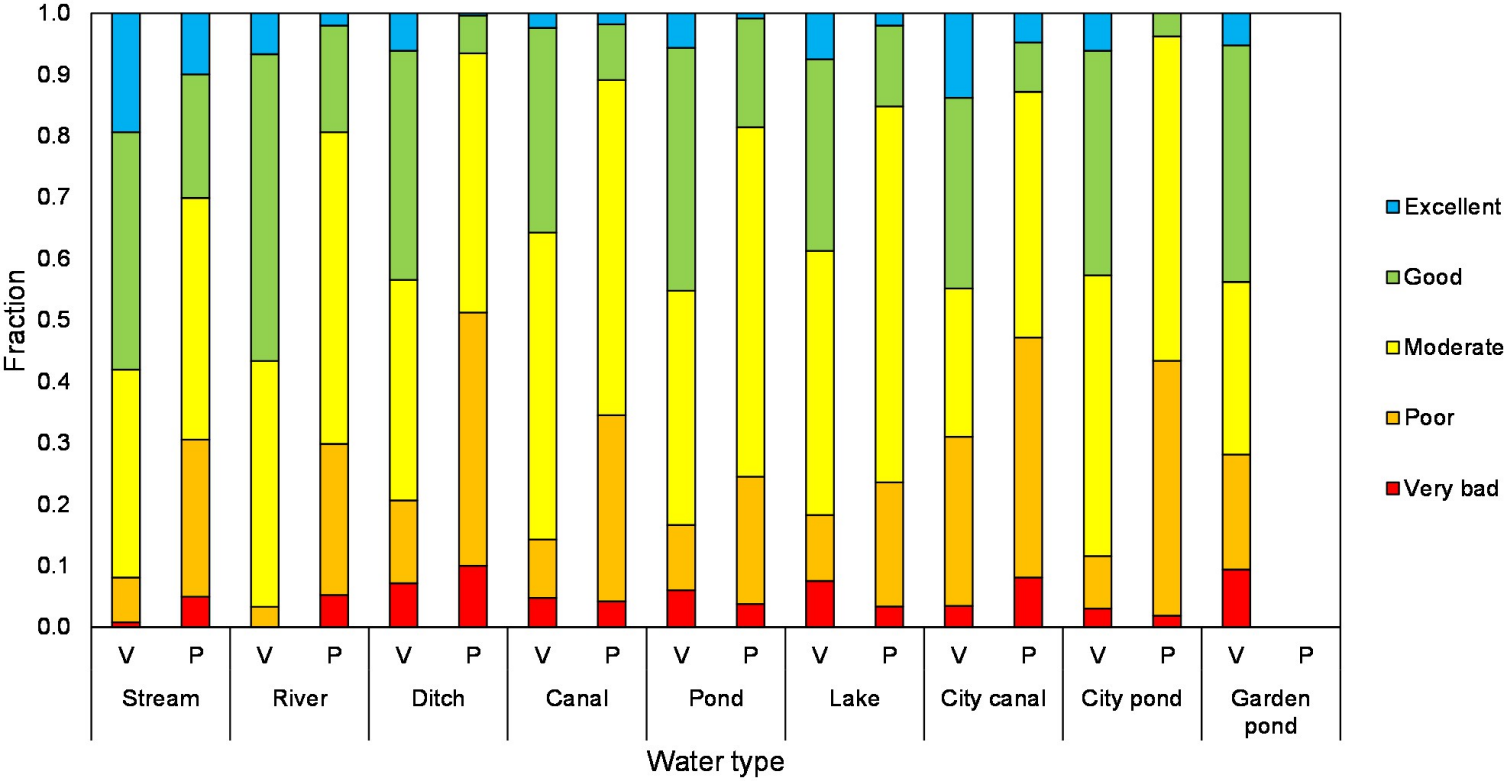
Distribution of water quality classes per water type as obtained from the volunteers (V) in the period 2018–2020 and professionals (P) in the period 2013–2019.

According to the WQ_cs_ scores, a relatively small proportion of the samples had a very bad or excellent water quality both for the citizen science dataset as well as for the professional dataset (Fig 5). In comparison to the professionals, the citizen science dataset contained locations with a good quality more frequently while professionals had larger fractions of moderate or poor quality. Approximately 30 to 60% of the samples collected by volunteers indicated a good or excellent quality while this was between 5 and 30% for the professionals (depending on the water type). In general, the proportion of waterbodies sampled by volunteers that had an excellent quality was below 10% except for running waters (19%) and city canals (13%). WQ_cs_ scores from garden ponds and city canals showed to have both a relatively large amount of good and excellent quality as well as poor and very bad quality. The number of samples with a moderate quality is limited.

The Fisher’s Exact Test indicates that volunteers had more observations than expected in particularly the good water quality class and to a lower extend in the excellent quality class compared to professionals. Additionally, volunteers had fewer observations than expected for the poor and moderate quality class while this was completely the reverse for the professionals (SII Appendix in S1 File).

## Discussion

The present study clearly indicates that only a very small fraction of the locations sampled by volunteers were in close vicinity of a location where professionals took samples in the last 10 years. Since the water system in the Netherlands is highly complex and branched, a distance of less than 50m between sampling points may still indicate physically separated waterbodies. Therefore, the comparisons show that volunteers indeed gather information on water quality at other geographical locations than professionals. Furthermore, volunteers more frequently investigated waters that are usually understudied by regional water authorities such as urban waters [62]. This study also shows that both professionals and volunteers collected animals mostly in spring, however, professionals started collecting data nearly two months earlier than volunteers. The peaks in number of samples in June in the volunteers data, are probably due to a yearly campaign of an NGO that invites children, parents and grandparents to collect water animals. Thus, both on the spatial and the temporal scale, the citizen science project yielded new data compared to the existing monitoring network carried out by professionals. This added value of citizen science has also been recognized in some previous studies like the monitoring of mosquitos in Germany [51], coastal mud crab in Finland [63], chemical water quality in England [64], and chemical water quality in combination with fish in temperate biomes [3]. Furthermore, monitoring by volunteers increases the number of observations [63] and is often regarded as a very cost-efficient way of gathering data for scientists [65–67]. Even though the monitoring by volunteers might seem cost-efficient, other hidden costs may add-up. For example, recruiting volunteers and keeping them interested in the long term requires staff with a dedicated amount of time and a specialization in marketing, education and communication [68, 69]. A citizen science platform like Waterdiertjes.nl cannot survive without sustained funding in the long run and this is a requirement to build up a long time series of data.

In 2020, twice as many observations were uploaded to the database than in previous years. Citizen science projects like the birdwatching project in South Africa [70] and City Nature Challenge in Tokyo [71] faced a lower degree of participation in 2020 due to covid-19. On the other hand, a European birdwatch project concluded that volunteers kept reporting birds but now more from home resulting in more observations in urban environments in the European countries UK, Italy and Spain and less in more remote areas [72]. The peak in the number of observations in June and autumn suggests that the covid-19 pandemic with many restrictions to minimize the spreading of the virus had a positive effect on people to explore their nearby environment and to participate in the project. Sampling waters in their own environment was still allowed as an activity for volunteers leading to a different use and experience of the blue-green space [73].

This study clearly shows that the monitoring by volunteers frequently include other water types than those routinely visited by professionals. Where professionals put a lot of effort in monitoring macroinvertebrates in streams, ditches and canals and to a lesser extend ponds, lakes and urban waters, volunteers focused their efforts more towards ditches, ponds, and especially to the urban environment. The focus on specific water types during special campaigns by NGOs may have led to more observations in these water types in the citizen database. It is well-known that macroinvertebrate community composition differs between water types [60] and thus sampling other water types by volunteers will generate more information about the regional quality of the aquatic environment for professionals.

Volunteers collected significantly less animals per sample than professionals. While professionals follow standard protocols (5m multihabitat sampling see e.g. [57]), it is very unlikely to expect volunteers will do the same since there are no instructions given to them with respect to sample size. Furthermore, equipment used by volunteers is frequently less sophisticated and presumably volunteers spent less time on the collecting process itself. Therefore, differences in tools, methods, available time and skills will contribute to the lower catches by volunteers. Furthermore, volunteers collected relatively more animals that were actively moving, probably because these animals attract their attention faster than those staying motionless or moving slowly. Professionals collected much more animals that live in and on (muddy) sediments while volunteers, on the other hand, caught more animals from the open water column and water surface. It seems, therefore, that volunteers incompletely sample the aquatic ecosystem and that they have little interest in sampling the soft sediments. As a result, this may hamper the added value of the data collected by volunteers to the professionals. Several studies have shown that training of volunteers may increase the accuracy and precision of citizen science phenological data [74, 75] and also specifically for aquatic macroinvertebrates [31]. However, a compulsory training usually comes at the cost of a lower degree of participation.

Many biological water quality assessment systems have been developed for specific water types like the BMWP for streams and rivers [40], and EcoFrame for lakes [45]. These systems require identification of macroinvertebrates down to the genus or species level, which is not realistic for untrained volunteers. The number of biological assessment systems that may be applied to all freshwater surface waters is still very limited. Here, a system is presented that can be used to assess the quality in a variety of aquatic ecosystems based on an identification level that doesn’t require trained amateurs or professionals but can be done by members of the general public. The quality of citizen science data is frequently questioned [76–79] and this is also relevant for the present citizen science project. Data provided on day of sampling and geographical coordinates is less prone to bias than collecting and identifying macroinvertebrates. Identification of the animals to higher taxonomic units, as is done in this project, certainly reduces possible faults and mistakes and this will contribute to a greater reliability of the data generated by the volunteers. Nevertheless, mistakes in identification are always possible and unavoidable, both for volunteers as well as for professionals. However, some taxa that might be difficult to distinguish from each other by unexperienced people like a swimming mayfly larva and a young damselfly larva, will not have a large impact on the calculated water quality score because both have the same indicator value. In addition, the reliability of the water quality outcome may also be increased when the total number of collected animals is taken into account. The higher the number of individuals included in the water quality calculation, the smaller the effect of minor errors in identification will be on the water quality score. Collecting a minimum number of animals by volunteers as is done in e.g. a Californian bio-survey [80] may increase the reliability of the quality score to a level comparable to professionals. Citizen science projects from other fields [31, 81, 82] show that such a professional level can be achieved. Volunteers in this project collected macroinvertebrates without strict protocols and this is frequently regarded as problematic for the quality of citizen science. However, lack of standardised sampling protocols and insufficient sample size is not unique to citizen science and is also problematic in regular sciences [83]. Despite these omissions, the information gathered by volunteers can still be very valuable for water managers. The volunteers’ observation that a species is present is more valuable than the notion of the absence a species since the latter is not a guarantee that the species is really absent. In the various biotic indices [39–42], the presence of listed key species is essential for the water quality assessment and not their absence. Thus, the volunteers’ registration of indicators of good or bad water quality can be valuable information for professionals.

The Dutch national Environmental Data Compendium classified the waters from the professionals based on macroinvertebrates and the professional assessment tool as 31% being excellent or good, 46% moderate, 21% poor and 1% very bad (PBL: https://www.clo.nl/en/indicators/en1438-quality-surface-water). This roughly corresponds with the general pattern in the citizen dataset (46% excellent or good, 37% moderate, 12% poor, 5% very bad). Interestingly, the water quality score (WQ_cs_) for the data of the professionals, was in general lower than the water quality score based on the data collected by volunteers. A possible explanation for this pattern could be that volunteers more frequently sampled smaller waterbodies that usually are richer in species [84] and have a higher water quality. Another reason could be that this apparently overestimated quality by the volunteers correlates with the much lower amount of soft sediment inhabiting organisms collected by the volunteers. Those animals, like worms and midges, are usually indicators of bad water quality. If they are not recorded, this obviously results in a higher water quality score. So the observed discrepancy in collected taxa between volunteers and professionals coincides with the differences in quality scores. Soft sediment inhabiting animals are part of the WQ_sc_ scores and, therefore, sampling protocols or the animals’ indicator values should be reconsidered for a better match between the volunteer and professional data and water quality scores.

Volunteers sampled a really different set of waterbodies than professionals. Until present, the information gathered by the volunteers is not used by the professionals, partially due to the discrepancy in methodology between volunteers and professionals. Professionals identify organisms preferably to the species level, while volunteers identify on a much higher taxonomic level. The associated water quality assessment systems used by professionals and volunteers, therefore, also differ. It seems that the information gathered by volunteers is not in the required format for immediate use by professionals. If this mismatch can be bridged in the future, the data gathered by the volunteers will be of much more value to the professionals. A possible approach could be to develop a quality assurance plan that describes defined methods to collect the data and that needs to be approved by the water authorities, as is the case in several citizen science projects in the USA [85]. This bridging is of utmost importance since citizen science is regarded essential for monitoring UN sustainable development goals [5–9] as well as for the implementation of the EU water framework directive [10–12].

In conclusion, information gathered via citizen science on biological water quality in the Netherland potentially adds to the information gathered by regional water authorities as volunteers collect data on other spatial and temporal scales. Volunteers however collect data in a different way and their inventory is less complete. New tools and processes need to be developed to make the gathered information more useful to professional water managers.

## Supporting information

S1 File (DOCX)
