# Supplementary material for: Monitoring biological water quality by volunteers complements professional assessments
Source: PLoS One. 2022 Feb 25;17(2):e0263899. doi: 10.1371/journal.pone.0263899 (PMC8880917; doi:10.1371/journal.pone.0263899)
Supplement: S1 File — (DOCX) [file pone.0263899.s001.docx]

Appendix SI. Standardised residuals from a Fisher’s Exact Test (Chi-square = 436.955, df = 8, p ≤ 0.001) to evaluate differences in sampling effort per water type between citizens and professionals. Negative values indicate that the number of observations was lower than expected while positive values indicate higher number of observations then expected. Colored cells indicate significant difference between observed and expected with red for lower and blue for higher expected counts than observed**.**

| Watertype | Volunteers | Professionals |
| --- | --- | --- |
| Streams | -8.7 | 7.6 |
| River | -2.1 | 1.8 |
| Ditch | 7.8 | -6.8 |
| Canal | -5.5 | 4.7 |
| Pond | 3.9 | -3.4 |
| Lake | -0.1 | 0.1 |
| City canal | -3.7 | 3.2 |
| City pond | 5.4 | -4.7 |
| Garden pond | 4.9 | -4.3 |

Appendix SII: Standardised residuals from Fisher’s Exact Test to evaluate water quality classes per water type and in total as obtained by volunteers and professionals. Negative values indicate that the number of observations was lower than expected while positive values indicate higher number of observations then expected. Colored cells indicate significant difference between observed and expected with red for lower and blue for higher expected counts than observed.

|  |  |  |  |  |  |  | Residuals |  |  |
| --- | --- | --- | --- | --- | --- | --- | --- | --- | --- |
| Type | Chi-square | df | p | Source | Very bad | poor | moderate | good | excellent |
| Streams | 51.051 | 4 | <0.001 | Volunteers | -2.0 | -3.9 | -0.9 | 4.2 | 3.1 |
|  |  |  |  | Profs | 0.5 | 0.9 | 0.2 | -1.0 | -0.7 |
| Rivers | 21.421 | 4 | <0.001 | Volunteers | -1.2 | -2.20 | -0.7 | 3.5 | 1.4 |
|  |  |  |  | Profs | 0.4 | 0.84 | 0.3 | -1.2 | -0.5 |
| Ditch | 340.029 | 4 | <0.001 | Volunteers | -1.4 | -7.5 | -1.4 | 9.6 | 4.6 |
|  |  |  |  | Profs | 1.4 | 7.4 | 1.4 | -9.4 | -4.5 |
| Canal | 23.401 | 4 | <0.001 | Volunteers | 0.2 | -2.4 | -0.4 | 4.6 | 0.3 |
|  |  |  |  | Profs | -0.0 | 0.5 | 0.1 | -1.1 | -0.1 |
| Pond | 64.570 | 4 | <0.001 | Volunteers | 1.0 | -2.4 | -2.6 | 4.0 | 2.7 |
|  |  |  |  | Profs | -0.9 | 2.1 | 2.2 | -3.5 | -2.4 |
| Lake | 28.241 | 4 | <0.001 | Volunteers | 1.5 | -1.6 | -1.8 | 3.2 | 2.2 |
|  |  |  |  | Profs | -0.8 | 0.9 | 1.0 | -1.8 | -0.5 |
| City Canal | 17.332 | 4 | <0.001 | Volunteers | -0.8 | -0.9 | -1.3 | 3.7 | 2.0 |
|  |  |  |  | Profs | 0.2 | 0.2 | 0.3 | -1.0 | -0.51 |
| City Pond | 45.628 | 4 | <0.001 | Volunteers | 0.2 | -2.5 | -0.3 | 1.9 | 0.9 |
|  |  |  |  | Profs | -0.4 | 4.5 | 0.6 | -3.4 | -1.6 |
| Total | 508.043 | 4 | <0.001 | Volunteers | 0.3 | -10.5 | -3.8 | 15.9 | 2.6 |
|  |  |  |  | Profs | -0.2 | 6.0 | 2.2 | -9.0 | -1.5 |
